# Pathophysiology, Evaluation, and Management of Edema in Childhood Nephrotic Syndrome

**DOI:** 10.3389/fped.2015.00111

**Published:** 2016-01-11

**Authors:** Demetrius Ellis

**Affiliations:** ^1^Division of Pediatric Nephrology, Children’s Hospital of Pittsburgh of UPMC, University of Pittsburgh School of Medicine, Pittsburgh, PA, USA

**Keywords:** edema, nephrotic syndrome, children/pediatrics, pathophysiology, management

## Abstract

Generalized edema is a major presenting clinical feature of children with nephrotic syndrome (NS) exemplified by such primary conditions as minimal change disease (MCD). In these children with classical NS and marked proteinuria and hypoalbuminemia, the ensuing tendency to hypovolemia triggers compensatory physiological mechanisms, which enhance renal sodium (Na^+^) and water retention; this is known as the “underfill hypothesis.” Edema can also occur in secondary forms of NS and several other glomerulonephritides, in which the degree of proteinuria and hypoalbuminemia, are variable. In contrast to MCD, in these latter conditions, the predominant mechanism of edema formation is “primary” or “pathophysiological,” Na^+^ and water retention; this is known as the “overfill hypothesis.” A major clinical challenge in children with these disorders is to distinguish the predominant mechanism of edema formation, identify other potential contributing factors, and prevent the deleterious effects of diuretic regimens in those with unsuspected reduced effective circulatory volume (i.e., underfill). This article reviews the Starling forces that become altered in NS so as to tip the balance of fluid movement in favor of edema formation. An understanding of these pathomechanisms then serves to formulate a more rational approach to prevention, evaluation, and management of such edema.

## Introduction

Edema is an essential clinical feature of the diagnosis of nephrotic syndrome (NS) of various etiologies. It is defined as a palpable swelling resulting from an accumulation of fluid in the interstitial fluid compartment. Massive generalized edema (anasarca) is common, especially in children with primary minimal change disease (MCD) and serves as the main clinical justification for hospital admission for “diuretic management.” In such children, the selective loss of large amounts of albumin in the urine leads to hypoalbuminemia and decreased plasma oncotic pressure favoring fluid sequestration in the interstitial fluid compartment, and secondarily triggers renal Na^+^ and fluid retention so as to preserve intravascular volume and blood pressure, hence preventing an “underfill” state. In contrast to MCD, in NS associated with glomerulonephritis the magnitude of the proteinuria is variable and reduction in GFR is common. Hence, in disorders, such as focal and segmental glomerulosclerosis, acute post-streptococcal glomerulonephritis, Henoch Schoenlein nephritis, lupus nephritis, and several other glomerulonephritides, intravascular fluid volume is typically normal or expanded because of inappropriately stimulated Na^+^ and fluid retention which together with decreased GFR results in an “overfill” state. The clinical distinction of these two predominant physiological states is critical to the proper management of edema in children with NS. However, the role of hypoalbuminemia has been questioned, and it is apparent that other mechanisms such as water retention and vascular permeability factors, and activation of channels that promote Na^+^ reabsorption, may also play an important role in edema formation in diverse causes of NS. Thus, multiple mechanisms acting simultaneously may explain the clinical variability in response to measures aimed at removing excessive fluid among individual children with NS. This has led to inconsistent or improper clinical evaluation and management of this relatively common disorder.

While the precise mechanism(s) of edema formation may be controversial and turn-around time of several laboratory tests used to confirm the underlying mechanism(s) may not be sufficiently rapid so as to influence acute management decisions, in most cases, there is adequate information to aid the clinical differentiation of the underlying basic mechanism of edema. This review discusses the pathophysiological alterations that favor edema formation in NS. This background then serves as the basis for developing selection criteria for hospital admission of children with NS who may benefit from edema fluid removal, establishing more standard guidelines for the clinical evaluation of nephrotic edema, and delineation of more uniform management guidelines aimed at providing effective management of the edema. Ultimately, the goal of therapy is to minimize the risk for hypovolemia, acute kidney injury (AKI), and other potentially serious complications ensuing from inappropriate diuretic regimens.

## Pathophysiology of Edema Formation in NS

Compared to adolescents and adults, neonates and younger children have a greater proportion of total body and interstitial (IS) fluid volume, which can double or triple because of edema related to NS (1). Nephrotic edema is a transudate with low protein concentration (<3 g/dL) and a few or no cells. In contrast to other forms of edema, it is pitting, tends to be generalized and is more prominent in dependent body areas in which capillary hydraulic pressure (P_CAP_) is high (ankles and feet), or in tissues with low resistance or low interstitial hydraulic pressure (P_IF_) (eyelids, gastrointestinal tract/abdomen, and scrotum).

It is apparent that while total body water remains constant during health, the fluid within each compartment is not static; there is continuous movement between compartments. The process of *diffusion* across cell membranes by far accounts for most fluid turnover (about 80,000 L/day in a 70 kg adult). By contrast, nephrotic edema represents net movement of water from the intravascular (IV) into the IS fluid compartment through the process of *filtration* across the capillary wall. Such filtration is dependent on the balance or net gradient, between the hydrostatic pressure and the oncotic pressure gradients across the capillary, as initially described by Starling in 1896 (2–4). Starling’s equation or “Law” was later modified as it became apparent that the net filtration is also determined by **Lp**, **S**, and **s**, as shown below:

$$\begin{array}{c} \text{Net filtration}=\text{LpS}\times (\Delta \text{ hydraulic pressure}-\Delta \text{ oncotic pressure)}\\ =\mathrm{LpS}\times [(\text{Pcap}-\text{ Pif})-s(\pi \text{cap}-\pi \text{if})] \end{array}$$

**Lp** is the unit permeability (known as porosity or “hydraulic conductivity”) of the capillary wall, which in the kidney refers to the glomerular capillary; **S** is the surface area available for fluid movement; **Pcap** and **Pif** are the capillary and interstitial fluid hydraulic pressures; **πcap** and **πif** are the capillary and interstitial fluid oncotic pressures; and **s** represents the reflection coefficient of proteins across capillary walls which ranges from 0 for vessels completely permeable to proteins and approaches a value of 1.0 in protein-free ultrafiltrate. The interstitial fluid oncotic pressure is derived primarily from filtered plasma proteins and to a lesser degree from proteoglycans in the interstitium.

Because of unique anatomy, physiological properties, and Starling forces of glomerular capillaries, the kidney plays a central role in total body fluid and electrolyte homeostasis as depicted elsewhere (5). Of note, the mean net ultrafiltration filtration pressure in glomerular capillaries is quite small and amounts to only 6–8 mmHg. Because normal glomerular capillaries are essentially impermeable to protein (so oncotic pressure in the filtrate is zero), this is a true ultrafiltrate with a high reflection coefficient (**s** is about 1.0). Several forces are altered in NS so as to favor net fluid filtration both in glomerular and in peripheral capillaries. These include reduction in **πcap**, increased **Lp**, elevation in **Pcap** depending on the underlying etiology of NS, increased **πif** and lymphatic vessel obstruction which may interfere with removal of filtered fluid thereby enhancing edema formation. Children with MCD tend to have more marked proteinuria and lower πcap than adults, while those with acute post-streptococcal glomerulonephritis tend to have Na^+^ retention and rise in Pcap, favoring edema formation.

**Figure 1:**
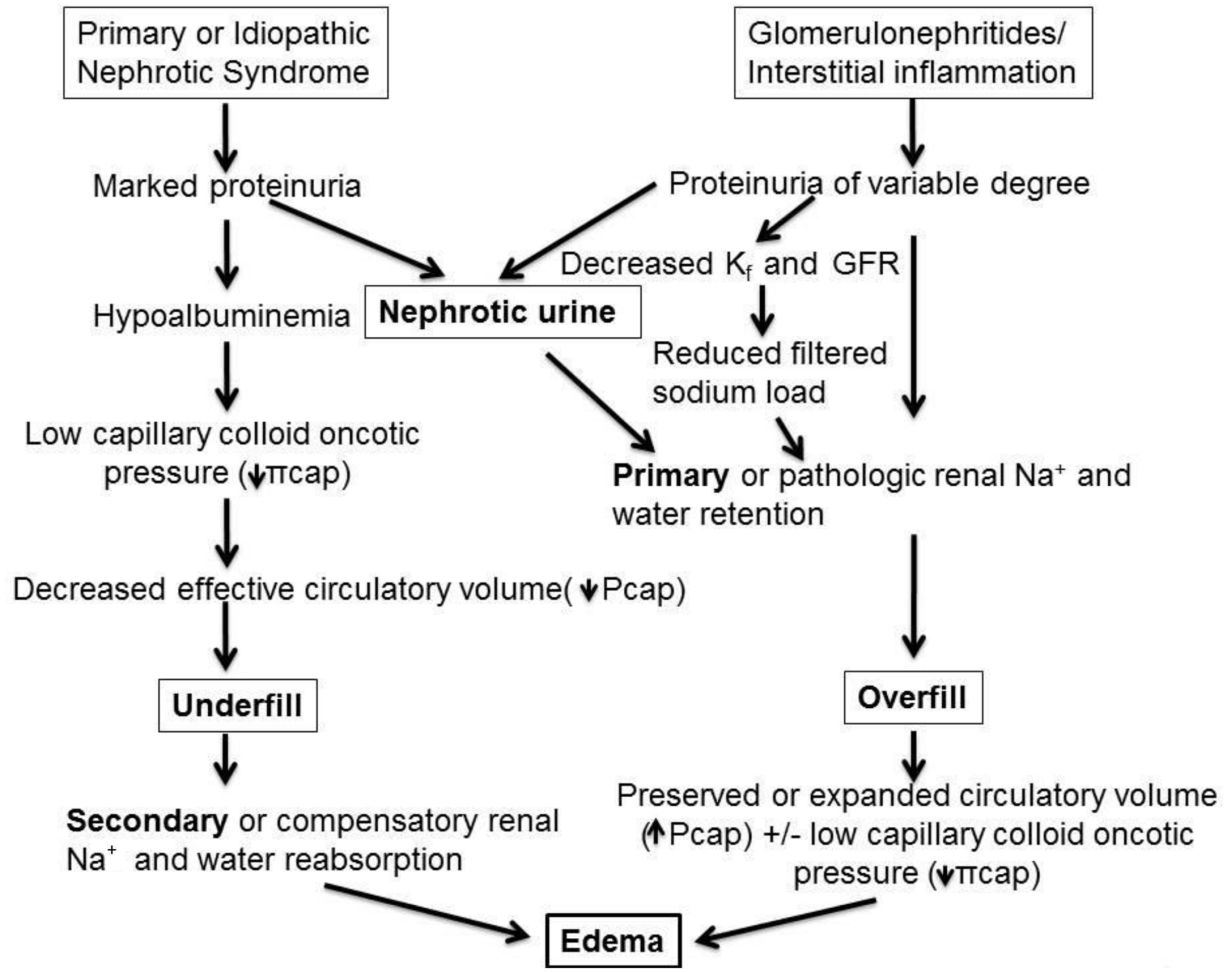
**Pathophysiology of edema formation in NS**. In disorders with massive proteinuria and marked hypoalbuminemia but minimal or absent renal inflammatory infiltrate, as in most children with minimal-change disease (MCD), the reduction in capillary colloid oncotic pressure (πcap) favors net fluid exit from the vascular to the interstitial fluid compartment thereby reducing effective circulatory blood volume, denoted as “underfill.” This then triggers secondary, or compensatory, Na^+^ retention and hemodynamic alterations aimed at achieving blood pressure homeostasis. By contrast, various glomerulonephritides and inflammatory renal disorders, such as acute post-streptococcal glomerulonephritis, may be associated with variable degrees of proteinuria and with pathologic release of mediators, which promote primary renal Na^+^ and water retention, as well as vasoconstrictive hormones that are released despite an intact or even expanded intravascular volume. These factors together with reduction in glomerular ultrafiltration coefficient (K_f_, or LpS in the Starling equation), lead to reduced glomerular filtration rate (GFR) and combine to further limit Na^+^ excretion, resulting in an “overfill” state and rise in capillary hydraulic pressure (Pcap). In turn, this causes net fluid accumulation in the interstitial fluid compartment. Note that nephrotic urine may be a common pathway for primary Na^+^ retention in both undefill and overfill disorders (refer to text and Tables 1–3 for the mediators sub-serving each of these main mechanisms of edema formation).

While mathematically and conceptually sound in providing a theoretical framework, direct measurement of Starling forces is complex and particularly challenging to assess in NS in which there are multiple alterations acting in concert to alter all components of Starling’s equation to different degrees in any given individual. For example, **Lp** can differ based on histopathological cause of NS, type and extent of elaboration of permeability factors that affect pore size and protein permeability, secretion of neuroendocrine hormones involved in preserving **Pcap**, and the concentration of plasma and interstitial albumin and other proteins that influence **πcap** and **πif**. Thus, in clinical practice, a glimpse of the basic operative mechanisms is often inferred by clinical assessment of hypovolemic symptoms or signs and by measurement of a limited number of blood and urinary conformational biochemical studies. Consequently, from a practical standpoint, two major mechanisms of edema formation are prevalent. First, in relation to edema associated with MCD or other non-inflammatory conditions resulting in massive proteinuria, an increase in transcapillary oncotic pressure gradient is the single most important driver of edema formation. This is because albumin which contributes 6 mmHg of oncotic pressure per g/dL, or, 24 out of 26 mmHg of normal plasma oncotic pressure, is markedly reduced in such disorders. According to the *underfill hypothesis* reduction in plasma oncotic pressure promotes net movement of fluid out of the intravascular compartment, leading to volume depletion, or underfilling (6–12). This then causes appropriate or compensatory physiological activation of several mechanisms ultimately resulting in secondary Na^+^ and fluid retention aimed at restoring intravascular volume and blood pressure. This is the most prevalent mechanism in edematous children presenting with NS. This is in contrast to the second mechanism of nephrotic edema, or *overfill hypothesis*, exemplified by acute post-streptococcal glomerulonephritis and other inflammatory proteinuric disorders in which the pathophysiological process activates several mediators sub-serving primary Na^+^ retention, resulting in intravascular volume expansion, increased capillary hydraulic pressure and edema. A schema depicting the two major mechanisms of nephrotic edema is presented in Figure 1.

## Controversy of Mechanisms of Nephrotic Edema

### Primary vs. Secondary Na^+^ Retention

While previous schemes of nephrotic edema depicted Na^+^ retention through distinct secondary (in underfill) and primary (in overfill) mechanisms, there is now compelling evidence that primary Na^+^ retention may occur by a mechanism common to all individuals with “nephrotic urine,” independent of underlying histology or blood volume status [see below, Ref. (13)]. Several studies implicate loss of plasmin and other serine proteases in the urine in the up-regulation of the epithelial sodium channel (ENaC) causing Na^+^ and fluid retention (see below). This is also highlighted in Figure 1.

### Blood Volume Status

Based on clinical observations and theoretical grounds, the long held prevailing opinion has been that children and adults presenting with NS edema, are in a state of intravascular volume depletion. Although a few studies support the presence of hypovolemia in adults with MCD compared to other histological forms of NS (14), and in some but not all children with MCD (15, 16), other investigations indicate that most adults and some children with NS of any cause have an adequate or expanded effective blood volume and do not exhibit orthostatic hypotension or other hypovolemic symptoms and signs (16–18).

To gain greater insight into the pathophysiology of edema in children with NS, Vande Walle et al. (19) studied children in early relapse of NS and found that they grouped into three presentations: (a) incipient proteinuria without edema, Na^+^ retention, and slightly elevated circulating aldosterone, increased renal plasma flow, and normal norepinephrine (NE) levels; (b) symptoms of hypovolemia, edema, Na^+^ retention, elevated plasma renin, aldosterone and NE levels, low atrial natriuretic peptide, and reduced GFR; and (c) edema, no hypovolemia, no active Na^+^ retention, normal plasma hormones, and normal GFR.

These data are very important because they indicate that there is both clinical and biochemical heterogeneity in children with NS. Distinguishing these fundamental mechanisms aids, the clinical prediction of safety and benefit of diuretic therapy: diuretics are well tolerated in children with primary renal Na^+^ retention but, if underfilling is the predominant mechanism, diuretic use can exacerbate hypovolemia and tissue hypoperfusion.

### Role of Hypoalbuminemia in Nephrotic Edema

Figure 1 highlights the role of hypoalbuminemia in children with nephrotic edema. However, with the exception of marked hypoalbuminemia (<2.0 g/dL) and plasma oncotic pressures below 8–10 mmHg, several clinical and experimental observations call into question the central role of hypoalbuminemia in the pathogenesis of nephrotic edema (8). For example, studies in analbuminemic rats show no significant change in transcapillary oncotic pressure gradient or tendency to edema compared to controls (20). Also, 81% of individuals with congenital analbuminemia (dysfunction of the albumin gene resulting in marked hypoalbuminemia) have little or no edema (21). It appears that in this disorder, **πcap** is partially compensated over time by other plasma proteins and may explain the parallel decrease in **πif**.

Other observations in experimental animals as well as in humans undergoing repeated plasmapheresis to intentionally produce modest reductions in serum albumin concentrations show that the transcapillary oncotic gradient is preserved; only with greater degree or more rapid achievement of hypoproteinemia does blood volume fall and edema develops (22, 23). This is because as plasma oncotic pressure falls, a parallel reduction in tissue oncotic pressure also occurs. Moreover, administration of albumin concentrates does not appreciably mobilize edema fluid in many nephrotic subjects, although Na^+^ excretion can increase transiently in some (24–26). Finally, patients with MCD who are treated with corticosteroids often undergo a diuresis and natriuresis well before the serum albumin concentration starts to rise; this finding suggests that correction of the hypoalbuminemia might not be essential in steroid-induced natriuresis (27). However, several of the experimental conditions investigating the role of albumin *per se* in the pathomechanism of nephrotic edema may not be appropriate models for this condition in humans in whom vascular permeability factors and cytokines may, in fact, play an important role in preventing the counterbalance of oncotic pressure gradient evident in congenital analbuminemia or in experimental plasmapheresis.

Despite the controversy surrounding the “central role” of hypoalbuminemia in the development of edema in NS, nearly all children with NS and edema have hypoalbuminemia and in the author’s opinion this controversy has little baring on the clinical evaluation and management of edema.

### Factors That Protect Against Edema Formation in NS

Because normally there is a small net pressure gradient favoring net filtration across capillaries, it might be expected that only a minor change in these hemodynamic forces would lead to edema. However, experimental and clinical observations indicate that there must be at least a 15 mmHg increase in the net pressure gradient favoring filtration before edema can be detected. With lesser reduction of this gradient, edema is unlikely to occur because of three compensatory factors (27, 28). First, experimental evidence indicates that there is increased lymphatic flow which, by bulk flow, will remove albumin as well, and help remove some of the excess filtrate (29, 30). Second, fluid entry into the interstitium will eventually raise the interstitial hydraulic pressure, thereby oppose filtration and interstitial fluid accumulation (28). Third, fluid accumulation in the interstitium simultaneously reduces interstitial oncotic pressure in subcutaneous tissue which in humans it is normally 12–15 mmHg (7). Thus, a gradual fall in plasma oncotic pressure in NS is associated with a parallel decline in interstitial oncotic pressure and rise in interstitial hydraulic pressure (7, 27), which minimizes the change in the transcapillary pressure gradients favoring net fluid movement out of the vascular space and results in relative preservation of plasma volume.

As a result of these compensatory physiological responses, there is usually little change in the transcapillary oncotic pressure gradient in children with NS, and therefore little tendency to plasma volume depletion, unless the hypoalbuminemia is severe. Similarly, as long as children with NS are not overdiuresed, plasma volume is typically preserved during diuretic therapy for edema removal.

## Volume Regulatory Hormones Sub-Serving Underfill and Overfill Mechanisms of Edema Formation in NS

In children who are hypovolemic or “underfilled,” there is interplay of several volume regulatory hormones and nephron channels listed in Table 1, which tend to attenuate the effect of vascular underfilling mainly through modulation of renal Na^+^ and fluid retention. Activation of the RAAS is an important mechanism in the majority of children presenting with nephrotic edema. It is notable that several of the hormones shown in Table 1, including AT II and anti-diuretic hormone (ADH), have dual Na^+^ or water retaining, and vasoconstriction properties, and are therefore very suitable in preserving systemic blood pressure in children with NS and reduced circulatory volume. Similarly, NE release in response to stimulation of low pressure cardiopulmonary receptors mediates neuronal control of renal tubular Na^+^ reabsorption (31). Edema may thus be viewed as a byproduct of these adaptive processes.

**Table 1:** **Contribution of volume and blood pressure regulatory hormones and channels which mediate or participate in renal Na^+^ and edema formation in NS**.

Hormones and channels	Function
RAAS activation	Direct stimulation of active Na^+^ reabsorption in the PCT by AT II; aldosterone-mediated Na^+^ retention
Non-osmotic ADH/vasopressin release	Water retention in CD, vasoconstriction
Norepinephrine (NE) release	α-Adrenergic stimulation of renal tubular Na^+^ reabsorption; vasoconstriction
Atrial natriuretic peptide (ANP) release	Promotes natriuresis and diuresis in DCT and CD, but tubular epithelium is resistant to these effects in NS
Urodilatin activation	Promotes natriuresis and diuresis in DCT and CD, but tubular epithelium is resistant to these effects in NS
Phosphodiesterase activation	Promotes degradation of ANP and urodilatin
Sodium-hydrogen exchanger 3 (NHE3) activation	Mediates Na^+^ reabsorption in PCT
Epithelial sodium channel (ENaC) activation by plasmin loss in nephrotic urine	Stimulates Na^+^ reabsorption in the DCT and CD
Sodium potassium ATPase (Na^+^/K^+^ ATPase) activation	Provides energy for pumps involved in active Na^+^ transport and facilitates peritubular uptake of Na^+^ by exporting Na^+^ out of cells in the anti-lumenal side of CCT

*RAAS, renin angiotensin aldosterone system; AT II, angiotensin II; Na^+^/K^+^ ATPase, sodium potassium adenosine tri-phosphatase; DCT, distal convoluted tubule; CD, collecting duct; PCT, proximal convoluted tubule; CCT, cortical collecting tubule*.

In addition, there may be several relatively unique pathological perturbations that promote edema formation in NS. Thus, children with acute post-streptococcal glomerulonephritis manifesting edema and hypertension often have increased circulating ANP and reduced ADH, plasma renin activity, aldosterone, and NE concentrations. Experimental models of unilateral NS or glomerulonephritis show increased Na^+^ reabsorption in the collecting tubules (26, 32), which is also the site of action of ANP and the related renal hormone urodilatin. Urodilatin is an ANP analog or isoform secreted by distal convoluted tubule (DCT) and collecting duct (CD) epithelium and exerts a paracrine function similar to ANP in promoting natriuresis and diuresis. In both experimental and human NS, a state of relative resistance to ANP and urodilatin has been observed (6, 33–35). This defect is due at least in part to urinary plasmin loss in individuals with NS which then stimulates phosphodiesterase activity, leading to more rapid degradation of the second messenger of ANP, cyclic guanosine monophosphate (cGMP), in the collecting tubules. Infusion of a phosphodiesterase inhibitor largely reverses this defect and restores the natriuretic response to volume expansion (33, 35).

Also, in many individuals with inflammatory forms of glomerulonephritis and NS there is activation of several tubular channels and transporters that promote Na^+^ reabsorption and edema formation. These include:

1. Increased activity of the Na^+^-hydrogen exchanger (NHE3) that mediates a large portion of proximal Na^+^ reabsorption (36, 37). However, as demonstrated in experimental unilateral NS, overall Na^+^ retention is influenced to a larger degree by mechanisms that promote Na^+^ reabsorption in the distal tubule (26).
2. Inactive plasminogen present in nephrotic urine is converted to active plasmin by the action of urokinase-type plasminogen activator. Cleavage and activation of γ subunit of the ENaC is then stimulated by the serine proteinase plasmin found in nephrotic urine, which may contribute to Na^+^ retention in the cortical collecting tubule (13, 38–41). This effect may be reversed by amiloride. This provides a common and potentially important mechanism by which filtered proteins cause primary Na^+^ retention regardless of the underlying histological form of NS or the child’s blood volume status. It also explains the clinical observation that spontaneous diuresis and edema improvement can occur in NS prior to a decrease in urinary protein excretion, despite ongoing hypoalbuminemia.
3. Increased activity of the Na-K-ATPase pump in the cortical collecting tubule but not in other nephron segments (42). This transporter provides the energy for active Na^+^ transport by pumping reabsorbed Na^+^ out of the cell and aiding it is uptake into the peritubular capillary. However, it is not clear if this represents a primary defect or if it is simply a secondary marker for increased Na^+^ transport at this site.

### Clinical Evaluation of the Predominant Mechanism of Edema formation in NS

Timely clinical assessment of hemodynamic aspects, including circulatory volume, is the key to determining management approaches to reduce edema in children with NS. This will help avoid exacerbation of the most common complication encountered in hospitalized children with NS and “underfill,” AKI (43), or hypertensive and pulmonary complications in those with “overfill.” Tables 2 and 3 summarize the typical clinical and laboratory features, which serve to distinguish children with NS with underfill or overfill physiology.

**Table 2:** **Mechanism of edema formation in nephrotic syndrome: “underfilling”**.

**Clinical characteristics**
Neuromuscular weakness, pallor, cool extremities, tachycardia, and other signs and symptoms of orthostatic hypotension, abdominal pain secondary to gut edema, abdominal compartment syndrome, or thrombosis of vena cave or renal veins
**Laboratory findings**
Reduced urine volume
FE_Na+_ < 0.2%
UK^+^/UK^+^ + Na^+^ > 60% (increased TTKG index)
Reduced urinary Na^+^ and high potassium concentration
Very low serum albumin (≤2 g/dL)
Low serum creatinine level
GFR > 75 mL/min/1.73 m^2^
Hemoconcentration
High circulating PRA, aldosterone, vasopressin, and norepinephrine
Low ANP concentration

*FE_Na+_, fractional excretion of sodium; UK^+^ and UNa^+^, urinary potassium and sodium concentrations; TTKG, transtubular potassium gradient; GFR, glomerular filtration rate; PRA, plasma renin activity; ANP, atrial natriuretic peptide*.

**Table 3:** **Mechanism of edema formation in nephrotic syndrome: “overfilling”**.

**Clinical findings**
Normal or elevated BP without tachycardia or orthostatic symptoms, and no signs to indicate distal extremity hypoperfusion
**Laboratory findings**
FE_Na+_ > 0.5% while on no salt restricted diet
UK^+^/UK^+^ + UNa^+^ < 60% (decreased TTKG index)
Hematuria and cellular casts
Serum albumin >2 g/dL
Elevated serum creatinine and BUN
GFR < 50 mL/min/1.73 m^2^
Decreased vasopressin
Low circulating PRA and norepinephrine
Low or normal plasma aldosterone
High ANP

*FE_Na+_, fractional excretion of sodium; UK^+^ and UNa^+^, urinary potassium and sodium concentrations; TTKG, transtubular potassium gradient; GFR, glomerular filtration rate; PRA, plasma renin activity; ANP, atrial natriuretic peptide*.

It should be noted that because Na^+^ retention occurs both in underfill and overfill states (Figure 1), it is not possible to use the FE_Na+_ to clinically differentiate primary from secondary Na^+^ retention in NS. Also, measurement of several hormonal markers shown in Tables 2 and 3 are not readily measured in hospital laboratories and may not be clinically useful in confirming the underlying operative mechanism or influence point-of-care decisions. However, an increase in RAAS and circulating aldosterone effect can be inferred on the basis of more readily measured values such as an increased transtubular potassium gradient (TTKG) index or, UK^+^/UK^+^ + UNa^+^, which is observed in hypovolemic children and not when blood volume is preserved. Similarly, a TTKG index below 60% along with FE_Na+_ above 0.5%, and normal or suppressed plasma aldosterone concentrations, highly implicate a primary Na^+^ retention mechanism leading to increased intravascular volume (i.e., overfill) (10, 19). This also suggests a primary role of aldosterone in the intrinsic activation of Na^+^/K^+^ ATPase in the cortical CD. Such children may benefit from diuretic use. By contrast, diuretic use in children with NS and secondary Na^+^ retention triggered by hypovolemia or circulatory insufficiency may have serious deleterious consequences (see below).

## Management of Nephrotic Edema

Before reviewing the management of edema in children with NS it is worth noting the change in the incidence of known clinical complications of NS that may relate to edema or its improper medical management. In a recent study involving hospital discharges of 4,701 children admitted with NS, Rheault et al. found that the frequency of infectious and thromboembolic complications has not changed much over the past 10 years; however, the incidence of AKI had increased from 3.3 to 8.5% (158%) over the period of 2001 and 2009 (43). The increasing use of nephrotoxic medications such as calcineurin inhibitors and angiotensin converting enzyme/angiotensin receptor blockers to co-manage steroid dependent and steroid resistant NS may be partly responsible for this trend. However, aggressive diuresis in children not recognized as having intravascular volume depletion (underfilling) may enable progression from incipient AKI to established AKI. Furthermore, prevention of AKI is of great importance because it may be a precursor to future development of chronic renal injury and hypertension. Inappropriate diuresis may also promote a thrombotic tendency in this disorder (44–47). Consequently, children with “underfill” physiology may benefit first by circulatory volume expansion using salt-poor albumin infusions, and delayed start of diuretics until after restoration of tissue perfusion is achieved.

## Non-Pharmacological Measures

Apart from managing the underlying condition leading to NS according to established guidelines (48–51), Table 4 summarizes other basic aspects of care. Because Na^+^ and fluid retention is a fundamental feature of all causes of NS and because treatment regimens that include corticosteroids tend to enhance this effect, all children presenting with edema are counseled on dietary Na^+^ restriction (35 mg Na^+^/kg/day, or approximately1.5 mEq/kg/day) and are monitored for clinical signs of hypovolemia (52). Fluid restriction is usually self-limited in children who adhere well to Na^+^ restriction, and it is not recommended in children managed in the outpatient setting.

**Table 4:** **General aspects of management of edema in children with NS**.

**Avoid NSAID use**
Avoid placement of deep lines to prevent thromboembolic events
Reduce dietary salt
No fluid restriction unless brisk diuresis is achieved
Insure adequate nutrition
Monitor urine output, renal function, electrolytes, serum albumin, body weight, and vital signs
Elevate extremities or use compression stockings when ambulating; water immersion is helpful but impractical
Avoid ACE inhibitors as remission can occur in many children with corticosteroid monotherapy

Attention to nutrition is very important particularly in conditions associated with massive proteinuria, such as Finnish type NS. Given the T1/2 of albumin of 21 days, when provided with adequate calories and amino acids, the liver can produce 200 mg albumin/kg/day to replace albumin catabolism or urinary loss. This normal synthetic function can double when the oncotic pressure in hepatic sinusoids falls as in the setting of NS. However, many children with NS are “picky eaters” and may have intestinal edema and abdominal pain, resulting in poor appetite as well as protein-losing enteropathy that may further compromise nutrition. In addition, the degree of proteinuria may be underestimated because of the large influence of the proximal tubule in albumin catabolism in NS. Thus, recycled amino acids are not used to replace albumin exclusively. Provision of supplemental calories, egg white protein, and nutritional supplements, such as Boost or Pediassure, may be helpful if clinically indicated.

Once a brisk diuresis is achieved, fluid intake may be limited to 2/3 of maintenance, or 1/2 or less of urine output, so as to produce the intended negative Na^+^ and fluid balance. Close monitoring of vital signs, fluid input and output, and of electrolytes is required in order to assure safety. Diuretic therapy should be temporarily discontinued if there is an unexplained decrease in urine output, elevation in serum creatinine or clinical manifestations of hypovolemia (e.g., weakness, orthostatic hypotension, and/or cool extremities) (52, 53).

## Pharmacological Management of Nephrotic Edema

### Albumin Infusion

In hospitalized children with nephrotic edema, excessive fluid can usually be removed, relatively safely, without exacerbating volume depletion (refer to Table 5). This is often accomplished through the combined administration of salt-poor, or, 25% albumin (SPA) to facilitate reabsorption of IS fluid, thereby supporting plasma volume and diuretics to enhance fluid removal. The more available 5% albumin solution can increase blood volume but does not raise oncotic pressure, while it delivers fivefold higher Na^+^ for each gram of albumin infused. By expanding plasma volume, albumin infusion suppresses vasopressin release induced by hypovolemia, thereby increasing water diuresis and improvement in hyponatremia. Albumin infusion is associated with more profound diuresis, at least in a subpopulation of pediatric patients with NS (54–57), particularly those with reduced effective arterial blood volume. For example, in one series of children with nephrotic edema and fractional excretion of Na^+^ (FE_Na+_) of <0.2% suggesting reduced effective arterial blood volume were treated with diuretics plus albumin infusion (52). Their diuresis rivaled that obtained by diuretic monotherapy in children with FE_Na+_ > 0.5% suggesting circulatory volume sufficiency or expansion (58).

**Table 5:** **Albumin infusion in the management of edema in NS**.

**Dosing of salt-poor albumin (25% SPA, or, 25 g/100 mL)**
0.5 g/kg infused over 1-h, 2–3 times daily. Slower infusion rates may enhance equilibration of albumin between the intravascular and interstitial fluid compartments, thereby undermining fluid mobilization and removal. Larger dosages may be more effective but may cause acute volume expansion and pulmonary congestion
**Indications**
Tense ascites with abdominal compartment syndrome limiting diaphragmatic excursion, lymphatic flow, and venous return
Severe pleural effusions compromising breathing
Oliguria with incipient acute kidney injury (AKI)
Marked eyelid edema compromising vision
Severe scrotal or labial edema, risking skin breakdown
**Precautions**
Expensive
Low supply
Obtained from multiple blood donors risking viral transmission, tissue allosensitization, etc.
Pulmonary edema

Table 5 lists specific indications for albumin infusion recommended by the author. Caretakers should also carefully weigh the precautions or potential drawbacks to such therapy, and consider avoiding pharmacotherapy for edema particularly if prompt remission of NS is anticipated. Notably, cosmetic effects related to edema are not an indication for diuresis in children with NS.

### Non-Protein Colloid Alternatives

The clinical utility of non-albumin colloids, such as (hyperoncotic 12% dextran solution given at 1.2–1.8 g/kg body weight daily as a single dose or on 3–5 consecutive days), was previously investigated in children with nephrotic edema (59). The precise mechanism of action has not been clarified. However, despite being effective in achieving a brisk diuresis at a lower cost than albumin, dextran use has not gained clinical favor because of safety concerns including increased blood pressure, headache, gastrointestinal discomfort, pain upon tissue infiltration, and bleeding diathesis with epistaxis. The clinical response to other non-protein colloid alternatives such as gelatin and hydroxyethyl starch to induce diuresis in nephrotic edema has not been investigated.

### Angiotensin Inhibition

Nearly, all children with chronic proteinuric disorders receive an ACE inhibitor or an angiotensin II receptor blocker as adjunctive synergistic drugs aimed at averting progressive renal injury caused by the underlying renal disorder. Such agents also exert a significant anti-proteinuric action and rise in serum albumin concentration which enhances the response to diuretics. However, the author recommends not using such agents in children with marked proteinuria because they tend to exaggerate hyponatremia and increase the risk of AKI because of lowering of systemic and intra-glomerular pressure. Although these agents serve as anti-proteinuric agents regardless of the underlying etiology of chronic proteinuria they are of little benefit in the acute management edema or if prompt remission of proteinuria is anticipated after staring steroids, as in the case of childhood MCD.

## Diuretic Management of Nephrotic Edema

Recognizing that in the majority of children nephrotic edema is the result of appropriate compensatory physiological mechanisms aimed at restoring diminished circulatory volume and tissue perfusion, use of diuretics to remove edema fluid should be undertaken with great caution so as not to interfere with adaptive responses (refer to Table 6).

**Table 6:** **Diuretics used to manage edema in children**.

Diuretic class, name, mechanism, and site of action	Bioavailability % PO/IV ratio		Onset of action (min) PO/IV	Duration of action (h)	Dosing
**Loop diuretics**					
Furosemide	60	1.5	40/5	6	Neonates: p.o. 1–4 mg/kg/dose, 1–2×/day iv/im 1–2 mg/kg/dose q 12–24 h
Bumetanide	85	1	40/5	4	Children: p.o./iv/im 1–2 mg/kg/dose q 6–12 h
Torsemide, ethacrynic acid					<6 months: p.o./iv/im 0.05–0.05 mg q 24 h
Inhibit the Na^+^/K^+^/2Cl^−^ cotransport system in the thick ascending limb of Henle’s loop (ALH)					>6 months: p.o./iv/im 0.015 mg/kg q 24 h; max. 0.1 mg/kg/dose
**Thiazide diuretics**					
Chlorothiazide	11–20		120	24	<6 months: p.o. 20–40 mg/kg/day divided bid iv 2–8 mg/kg/day divided bid
Hydrochlorothiazide	60–75		120	12–24	>6 months: p.o. 20 mg/kg/day divided bid iv 4 mg/kg/day
Inhibit NaCl cotransport in the early distal convoluted tubule (DCT)					<6 months: p.o. 2–3.3 mg/kg/dose divided bid >6 months: p.o. 2 mg/kg/day divided bid
**Thiazide-like**					
Metolazone	40–60		60	24	Children: 0.2–0.4 mg/kg/day divided q 12–24 h
Similar to thiazides but also proximal tubular inhibition of sodium uptake					

With the possible exception of children with inflammatory glomerulonephritis, nephrotic edema and coexisting hypertension or clear evidence of “overfilling,” the author rarely recommends diuretic use in the outpatient setting. This is because of the potentially catastrophic risks of diuretic use in NS, such as thrombosis and thromboembolism, AKI, and electrolyte imbalance. By contrast, concurrent use of diuretics and salt-poor albumin or diuretic monotherapy is often utilized to manage edema in the inpatient setting.

Many children with NS respond well to loop diuretics, although there is generally lesser natriuresis than when such diuretics are utilized to manage edema associated with other medical disorders (60, 61). Experimental studies in drug-induced NS suggest that the loop of Henle may be relatively resistant to loop diuretics (62). Several factors are thought to play an important role in inducing this relative diuretic resistance:

1. Because most diuretics are highly protein-bound, they tend to become trapped within the vascular compartment, thereby maximizing their rate of delivery to the kidney. In NS however, the degree of protein binding is reduced due to hypoalbuminemia, resulting in a larger extravascular space of distribution and diminished rate of delivery to the kidney (60, 61).
2. In order to function, loop diuretics must exit the vascular capillary, traverse the interstitium, enter the tubular epithelial cell and be secreted into the tubular lumen where they block Na^+^, chloride, and other transporters. Typically in NS, there is expansion of the renal parenchymal interstitial fluid compartment which reduces peritubular diuretic uptake.
3. Some of the diuretic that enters the tubular lumen is bound to filtered albumin and rendered inactive (63, 64). In experimental *in vivo* microperfusion of the loop of Henle, the addition of albumin to the perfusate in a concentration similar to that seen in the tubular lumen in NS diminishes the response to intraluminal furosemide by about 50% (63). However, it is uncertain if this mechanism is important in humans. In one study of seven patients with NS, blocking of albumin binding to furosemide by the administration of sulfisoxazole had no effect on the diuretic response (65).

In clinical practice, this diuretic resistant state can be partly overcome by administering a higher diuretic dosage in subjects with nephrotic edema compared with other edematous disorders (19). In younger children, furosemide is most often used to manage nephrotic edema. It is infused at 0.5 mg/kg/dose every 8–12 h, given at the start or at 1 h after administration of SPA. Also, a modest increase in urine Na^+^ excretion and volume has been reported in adults with marked hypoalbuminemia by infusing a solution consisting of furosemide added to SPA. In theory, this approach aids trapping of the diuretic within the vascular compartment, thereby increasing the rate of loop diuretic secretion into the tubular lumen (66).

A recent review of loop diuretics in managing nephrotic and other forms of systemic edema indicates several advantages of bumetanide, including greater bioavailability than furosemide as well as the convenience of 1:1 intravenous to oral conversion (67). However, because of high potency at low dosages this agent is somewhat difficult to titrate in smaller sized children. Because of a short half-life, all loop diuretics require multiple dosing. Ethacrynic acid is reserved for children with sulfa allergy. While popular in adults, there is limited experience with torsemide use in children.

In children who do not respond adequately to loop diuretics, it is advisable to add a thiazide type diuretic in order to achieve diuretic synergy by way of sequential nephron blockade. Chlorothiazide can be given orally or intravenously and is particularly useful in small sized children or if gastrointestinal absorption is compromised. For oral use in children over 5 years old, the author prefers short-term use of metolazone (Zaroxolyn), a long acting thiazide-like diuretic, which is secreted in the proximal tubule and has a plasma half-life of 36-hours; it is given at a dosage of 2.5–5.0 mg once daily. Like most loop diuretics, metolazone is also highly protein bound and, therefore, it is not dependent on normal GFR for it to be effective. When this is combined with bumetanide and SPA, a brisk diuresis is usually achieved.

Although ENac channel activation has been implicated in Na^+^ retention in NS of diverse etiologies, the efficacy of blocking this channel by amiloride is believed to be low because of the relatively small amount of Na^+^ arriving at the DCT. However, amiloride and other potassium sparing diuretics, such as spironolactone (1.25 mg/kg/dose), may be utilized in conjunction with loop diuretics.

## New Directions in the Management of Nephrotic Edema

### Aquaretics

Aquaretics are a newer group or class of diuretics which unlike conventional diuretics produce solute-free diuresis, or aquaresis. As discussed above, studies in untreated children with NS and underfill physiology have shown increased plasma and urinary concentrations of vasopressin or ADH (19). This together with presence of hyponatremia that is frequently found in these children is highly suggestive of water retention in excess of Na^+^ retention. These observations also apply to adults with NS (68–70) and provide the rationale for aquaretic use to manage nephrotic edema. A case report and early clinical trials (71) suggest an efficacy of brief courses of vasopressin 2 receptor anatagonists, such as tolvaptan, or of somatostatin (Octeotide), in inducing aquaresis by inhibiting their common intracellular mediator, cAMP, thereby decreasing aquaporin channel insertion in the renal CD epithelium and abrogating anti-diuresis.

Urea channel inhibitors provide another newer approach to aquaresis (72–75). Use of these agents relies on the fact that humans and other mammals consuming a high protein diet generate nitrogen which is then excreted by the kidneys in the form of urea. The large amount of urea filtered at the glomerulus tends to promote an osmotic diuresis. To attenuate such diuresis the kidney has “UT-A” (coded by the SLC14A2 gene) and “UT-B” (coded by the SLC14A1 gene) channels, which aid the accumulation of urea in the renal medullary interstitium. This action osmotically balances the urea in the CD lumen, thereby preventing urea-dependent osmotic diuresis that would otherwise occur. Li et al. have identified a compound that inhibits the UT-B urea channel (76). Compounds that inhibit the UT-A channels could be potentially more useful as aquaretic agents by blocking both the source of urea in the inner medulla (UT-A1 and UT-A3 channels in the inner medullary CD) and the countercurrent exchanger in the vascular bundles (UT-A2). Also, UT-A inhibitors are predicted to have fewer adverse effects, since UT-A’s only known physiological function is in the kidney, whereas UT-B inhibitors could cause hemolysis and other systemic adverse effects.

Like vaptans and somatostatin, urea channel inhibitors are of potential benefit in the treatment of hyponatremic disorders but may also be of benefit in managing edema associated with NS, particularly if excessive water retention is suspected on clinical evaluation. As with diuretics, aquaretics should only be considered in children with sufficient effective circulatory volume.

### Other Potential Therapies of Nephrotic Edema

Identification of molecules that initiate proteinuria or directly contribute to edema formation offers the possibility of modulation of a greater number of potential targets so as to neutralize their detrimental effects. Currently, this area of research is in its infancy.

## Author Contributions

DE was responsible for the concept, literature review, and manuscript preparation.

## Conflict of Interest Statement

The author declares that the research was conducted in the absence of any commercial or financial relationships that could be construed as a potential conflict of interest.
